# Vitamin B_12_ status in women of childbearing age in the UK and its relationship with national nutrient intake guidelines: results from two National Diet and Nutrition Surveys

**DOI:** 10.1136/bmjopen-2016-011247

**Published:** 2016-08-12

**Authors:** Nithya Sukumar, Antonysunil Adaikalakoteswari, Hema Venkataraman, Hendramoorthy Maheswaran, Ponnusamy Saravanan

**Affiliations:** 1 Warwick Medical School, University of Warwick, Coventry, UK; 2 Academic Department of Diabetes and Metabolism, George Eliot Hospital, Nuneaton, UK

**Keywords:** NUTRITION & DIETETICS

## Abstract

**Objective:**

To assess serum B_12_, folate and the associated homocysteine (Hcy) levels among women of childbearing age in the UK and examine their association with dietary intake in relation to the UK Recommended Nutrient Intakes (RNIs) for B_12_ and folate.

**Design:**

Cross-sectional study.

**Setting:**

Data from two publicly available National Diet and Nutrition Surveys (NDNS 2000/2001 and 2008/2012) were used. These were population-based surveys of randomly selected samples of adults which were carried out in their households.

**Participants:**

Women of childbearing age (aged 19–39 years), representative of the UK population. Those who were pregnant or breastfeeding were excluded.

**Outcome measures:**

The associations between micronutrient intakes and blood levels of B_12_, folate and Hcy were assessed by correlation and stepwise linear regression. B_12_ intake was divided into quintiles and plotted against blood B_12_ and Hcy concentrations to determine the threshold of any associations.

**Results:**

299 women from the first NDNS cohort had complete intake and biomarker data. The prevalence of serum vitamin B_12_ (≤150 pmol/L) and serum folate (≤10 nmol/L) deficiency and hyperhomocysteinemia (≥12 µmol/L) was 12.4%, 6.4% and 21.2%, respectively, despite seemingly adequate B_12_ intakes (median 3.8 μg/day, 96% consumed more than the UK RNI of 1.5 μg/day). B_12_ concentrations increased across all quintiles of intake with serum levels in quintiles 4 and 5 (median intake 4.9 and 7.1 μg/day, respectively) significantly higher than quintile 1. However, Hcy concentrations levelled off between quintiles 4 and 5. Comparison of micronutrient intake between the two surveys found that folate intake has reduced in the more recent cohort.

**Conclusions:**

The UK RNI for B_12_ intake should be increased for women of childbearing age with intakes of around 5–7 μg/day likely to be associated with stable biomarker levels. B_12_ levels should also be measured in women preconceptionally or in early pregnancy given the high rates of deficiency.

**Strengths and limitations of this study**

- Two publicly available data sets from the British National Diet and Nutrition Surveys (NDNS) were used to investigate the association between dietary intake of B_12_ and folate and their blood concentrations in women of childbearing age.
- The women sampled in the surveys were representative of UK adults; therefore, the findings can be generalised to the wider UK population.
- Information about the participants, including demographics, dietary intakes, blood results and anthropometry, was used for detailed analysis of the outcomes of interest.
- The availability of two separate NDNS data sets allowed comparison of micronutrient intakes over the last 15 years in young women.
- Blood micronutrient concentrations were not analysed in the more recent survey. Therefore, the associations observed in the first survey could not be replicated.

## Introduction

Vitamin B_12_ (B_12_), also known as cobalamin, is a micronutrient essential for cellular growth, differentiation and development.^1^ Along with folate, B_12_ is necessary for the synthesis of DNA, RNA, lipids and protein, and an essential step in this process is the conversion of homocysteine (Hcy) to methionine.^2^ Therefore, a deficiency of either B_12_ or folate leads to increased Hcy which can have significant clinical implications such as cardiovascular disease and atherosclerosis in adults.^3^ ^4^

During pregnancy, low circulating levels of B_12_ or folate have been associated with complications such as neural tube defects (NTDs),^5^ spontaneous abortion,^6^ premature birth^7^ and possibly low birth weight.^8^ Mandatory folic acid fortification in North America has resulted in reduction of NTD by over 40% in the last 10 years,^9^ but there has been a tripling of the condition attributable to B_12_ deficiency during the same time frame in this population.^10^ Maternal hyperhomocysteinemia has been linked to early pregnancy losses,^11^ pre-eclampsia^12^ and small for gestational age babies.^13^ Although the exact mechanisms are not known, some of these effects may be mediated by vascular compromise to the fetus and insufficient placental development which occurs in the early embryonic period.^14^ As around half of the pregnancies are unplanned, even in developed countries,^15^ there is increasing attention on optimising the nutrient status of women in the periconceptional period.

B_12_ deficiency is prevalent during pregnancy as shown by a large systematic review recently conducted by our group.^8^ We showed that the rates were in the order of 20–30% in all three trimesters across the world, with particular high rates in studies from the Indian subcontinent, Eastern Mediterranean and South American regions.^16–18^ A longitudinal study has shown that cobalamin levels can decrease by around 10–20% from preconception to the first and second trimesters, respectively.^19^ However, there are no studies in the UK that assessed the B_12_ intake or circulating levels in women of reproductive age prior to conception.

The UK Department of Health (DOH) has stipulated that the Recommended Nutrient Intake (RNI) for B_12_ and folate in adults is 1.5 and 200 μg per day, respectively, with no different recommendations for women (pregnant or otherwise) and the elderly.^20^ These recommendations were published in 1991 and state that the RNI for B_12_ ‘represent the level of intake considered likely to be sufficient to meet the requirements of 97.5% of the group’.^21^ The evidence for this RNI is from reports in the 1970s, which estimated the average requirement to prevent diet-related B_12_ deficiency and megaloblastic anaemia.^22–24^ B_12_ deficiency in these studies was arbitrarily defined as <150 pmol/L without use of any functional markers of B_12_ deficiency.^25^ ^26^ Thus, there are little contemporary data to support how accurately the recommended intakes correlate with serum values of B_12_ and functional indicators such as Hcy. The primary aim of this study is to determine the serum B_12_, folate and Hcy status of women of childbearing age in the UK and to assess the correlation between estimated B_12_ intake and blood concentrations of B_12_ and Hcy using the data from two British National Diet and Nutrition Survey (NDNS) in 2000/2001 and 2008/2012. The secondary aim is to compare the nutritional intake between the two NDNS cohort to provide more recent data on B_12_ intake.

## Methods

### Subjects

We used data collected from the two British NDNS between July 2000 and June 2001 and 2008–2012. The surveys provide detailed quantitative assessment of nutritional status and laboratory results of the participants. The methods used in the survey have previously been described in detail.^27–29^ The samples were made up of randomly selected adults aged 19–64 years living in private households, who were representative of the UK adult population. Their selection was done by a multistage, stratified, probability sampling with postal sectors as first stage units. If there were more than one adult in the same household, one was selected randomly. Women who were pregnant or breast feeding were excluded.

The selection of the participants included in our analysis is provided in online supplementary figures S1 and S2. For the first survey (2000/2001), the fieldwork was carried out over a 12-month period, with respondents being surveyed over four 3-month periods to account for seasonal variations in nutritional behaviour and content. Out of 3704 potentially eligible adults identified for the study, 299 women between 19 and 39 years of age (known as women of ‘childbearing age’ for the purpose of our analysis) with complete dietary and biomarker information were included in the final analysis. In an independent study,^30^ no evidence of was found of non-response bias in this NDNS data.

10.1136/bmjopen-2016-011247.supp2 Supplementary figures

The second survey (2008/2012) consisted of 2424 adults of whom 395 were women of childbearing age. Unfortunately, blood samples were only obtained from 157 of these women (ie, 39.7% of all females aged 19–39 years). The main reasons for this low return of blood samples were ‘no nurse visit’, ‘participant refused’ and ‘blood sample inapplicable’. Owing to the potential bias from a sample of women not representative of the larger NDNS cohort, we only included and compared the micronutrient intakes from the two surveys to assess the secular trend.

### Assessment of dietary intake

This has been described in detail elsewhere.^27^ Briefly, dietary assessment for each participant was done by a two-stage process: (1) two face-to-face interviews using computer-assisted personal interviewing methods, and (2) a 7-day dietary record using weighed food diaries. Each participant was provided with a set of Soehnle Quanta digital food scales and two recording diaries (for use at home and outside). Additional information was obtained about the use of medicines, vitamin and mineral supplements. Information provided in the food diaries was later used to determine nutrient intakes by linking to the Food Standards Agency nutrient database, which holds details for 56 nutrients for each of 6000 food codes.^27^

### Laboratory methods

Venous blood samples were taken at the non-fasting state by trained phlebotomists in participants' homes. Serum folate and B_12_ measurements were performed on the Abbott IMx semiautomated analyser, which uses microparticle enzyme immunoassay (MEIA) technology (z). Quality control consisted of an internal pooling of serum samples with each run for use as a drift control, and an external quality assessment by the UK National External Quality Assessment Service (NEQAS). Outlying results were defined as serum B_12_ level >1000 pmol/L and serum folate >60 nmol/L and were excluded. Plasma Hcy measurements were performed by the Abbott IMx assay on the IMx analyser. Quality control consisted of participation in an international external quality assessment scheme based in Denmark^31^ and by the manufacturer's QV samples.

### Statistical analysis

Statistical analysis was performed with SPSS V.22.0 (IBM SPSS Statistics for Windows, Version 22.0 (program). Armonk, NY: IBM Corp, Released 2013). For continuous variables (eg, mean B_12_/folate intakes and levels between RNI threshold groups), the Student’s t-test and for categorical variables (eg, B_12_ and folate deficiency rates), the χ^2^ test for independence or the Fisher's exact test was used. Stepwise multiple linear regression analysis with each of serum B_12_, folate and plasma Hcy as the dependent variable was done, with predictors entered or removed following the criteria: probability of F to enter ≤0.050, probability of F to remove ≥0.100. The regression models included the following covariates: age, body mass index (BMI), total cholesterol:high-density lipoprotein (TC:HDL) ratio, alcohol intake, smoking status, oral contraceptive use, vegan/vegetarian, serum folate and where appropriate daily folate intake (with supplements), daily B_12_ intake (with supplements) and serum B_12_ levels. Race was not included in the analysis as the majority of the participants (95%) were white. To determine the trend of B_12_ and Hcy across the spectrum of B_12_ intakes, we divided the cohort into quintiles of B_12_ intake. One-way analysis of variance (ANOVA) with Tukey's post hoc test was applied to compare the serum levels against the lowest quintile. Logistic regression was then done for incremental B_12_ intake thresholds of 0.25 μg to determine predictors of B_12_ deficiency and hyperhomocysteinemia. In order to estimate the prevalence of inadequate B_12_ intake in our population and observe how this was related to abnormal biomarker levels, we used the Estimated Average Requirement (EAR) cut-point method.^32^ The EAR was calculated as 1.25 μg/day based on a coefficient of variation of 10% below the UK RNI.^25^

The definitions for micronutrient deficiencies were as follows: serum B_12_≤150 pmol/L,^33^ serum folate ≤10 nmol/L^34^ and red cell folate <350 nmol/L.^34^ The upper limit of normal for plasma Hcy in non-pregnant adults aged 15–65 has been variably defined as 12 µmol/L in non-pregnant adults with folic acid fortification or supplementation^35^ and 15 µmol/L in those without fortification or supplementation, which applies to our population. However, Hcy levels above 10.7 µmol/L in women during preconception has been associated with pre-eclampsia, prematurity and very low birthweight infants if they become pregnant.^12^ In addition, other experts recommend <12 µmol/L for all adults.^36^ Therefore, we have presented results for 12 and 15 µmol/L and stated these clearly throughout.

## Results

From the NDNS 2000/01 cohort, 299 women of childbearing age had complete dietary and serum micronutrient level results. The demographics and clinical characteristics of these women are shown in table 1. Their mean age was 31.6 years and BMI 25.3 kg/m^2^. To determine how the B_12_/folate intakes women of childbearing age compared with older women from the same NDNS survey, we analysed these parameters in women aged 40–64 years. The younger women consumed significantly less B_12_ and folate (median 3.83 vs 5.16 and 237 vs 279 μg/day, respectively, p<0.001 for both) and consisted of more vegetarians/vegans (8.7% vs 4.2%, p<0.05) and less B_12_ supplement users (10.4% vs 16.2%, p<0.05). Folic acid supplement use was the same (data not shown).

**Table 1:** Demographics and B_12_ and folate intakes of women of childbearing age (NDNS 2000/2001 cohort)

Female 19–39 years	All subjects (n=299)
Age (years)	31.6±5.7
BMI (kg/m^2^)	25.4±5.2
Obesity, n (%)	46 (15.7)
Current smokers, n (%)	117 (39.1)
Regular alcohol drinkers, n (%)	267 (89.3)
Oral contraceptive use, n (%)	103 (34.4)
Ethnicity, n (%)	
White	283 (94.6)
Afro-Caribbean	3 (1.0)
Asian	8 (2.7)
Other	5 (1.7)
Vegetarians, n (%)	26 (8.7)
B_12_ supplement users, n (%)	31 (10.4)
B_12_ intake, diet only (μg/day)	3.82 (2.75, 5.02)
B_12_ intake, diet+supplements (μg/day)	3.83 (2.82, 5.20)
Folic acid supplement users, n (%)	32 (10.7)
Folate intake with supplements (μg/day)	237 (177, 315)

Continuous variables are mean±SD or median (IQR).

Categorical variables are n (%).

BMI, body mass index; NDNS, National Diet and Nutrition Surveys.

The blood levels of B_12_, folate and Hcy in women of childbearing age and according to categories of UK RNI for B_12_ intake (adequate/inadequate) are given in table 2. The median serum B_12_ concentration was 241 pmol/L. Overall, 12% of women were B_12_ deficient (<150 pmol/L), despite the median B_12_ intake of the deficient women being nearly two times the UK RNI (2.96 μg/day; data not shown). In total, 3.7% of the surveyed population had B_12_<150 pmol/L and Hcy>12 µmol/L, with a significantly higher proportion having the combination of abnormalities when their estimated was lower than the UK RNI (9.1% vs 3.5%, p=0.001). There is evidence that B_12_ levels <258 pmol/L may be indicative of B_12_ deficiency in certain individuals with concomitant elevation of Hcy and methylmalonic acid (MMA).^37^ In total, 44.0% of women had B_12_ levels in this borderline range of 150–258 pmol/L. In this subgroup, mean Hcy levels were significantly higher than the group with B_12_>258 pmol/L (10.4 vs 9.2 µmol/L, p=0.02) despite similar folate levels (21.4 vs 22.0 nmol/L, p=NS).

**Table 2:** Comparison of B_12_, folate and plasma homocysteine concentrations in women according to the UK RNI for vitamin B_12_ intake

Female 19–39 years	All subjects	UK RNI (μg/day)		p value*
		<1.5	≥1.5	
Number (%)	299 (100)	11 (3.7)	288 (96.3)	
B_12_ intake, diet only (μg/day)	3.82 (2.75, 5.02)†	1.29 (0.64, 1.46)	3.86 (2.86, 5.09)	<0.001
B_12_ intake, diet+supplements (μg/day)	3.83 (2.82, 5.20)	1.29 (0.98, 1.46)	3.92 (2.88, 5.32)	<0.001
Serum B_12_ (pmol/L)	241 (188, 324)	169 (153, 256)	244 (189, 325)	0.05
B_12_ deficiency (<150 pmol/L), n (%)	36 (12.0)	2 (18.2)	34 (11.8)	NS
Serum folate (nmol/L)	19.5 (14.1, 26.7)	14.3 (13.6, 21.3)	19.7 (14.2, 27.0)	NS
Serum folate deficiency (<10 nmol/L), n (%)	18 (6.1)	0 (0)	18 (6.3)	NS
Red cell folate (nmol/L)	584 (473.9, 750.6)	460 (372, 739)	585 (478, 751)	NS
Red cell folate deficiency (<350 nmol/L), n (%)	13 (4.4)	2 (18.2)	11 (3.8)	NS
Hcy (μmol/L)	9.4 (9.1, 9.8)	11.9 (9.6, 14.3)	9.2 (7.8, 11.4)	<0.05
High Hcy (>12 μmol/L), n (%)	62 (21.2)	5 (50)	57 (20.1)	<0.05
High Hcy (>15 μmol/L), n (%)	24 (8.2)	2 (20)	22 (7.8)	NS

*Comparison between lower and higher B_12_ intake groups. For categorical variables, Student’s t-test was used (after log transformation); for continuous variables, Fisher’s exact test was used.

†Median, 25–75th centile in parentheses (all such values).

Hcy, homocysteine; NS, not significant; UK RNI, UK Recommended Nutrient Intake.

The plasma Hcy concentrations were higher in the lower intake group (11.9 vs 9.2 µmol/L, p<0.05) although hyperhomocysteinemia (Hcy>12 µmol/L) was present in 20% of women with apparently adequate B_12_ intake (table 2). Serum and red cell folate deficiency rates were 6.1% and 4.4%, respectively, in the whole population. There were no differences in the folate deficiency rates between above and below the UK RNI B_12_ intake groups. In total, 34.4% of the women were taking the oral contraceptives and their B_12_ values were lower than those who were not (median 211.5 vs 267.5 pmol/L, p<0.001).

In total, 8.7% of women in the whole cohort were vegetarian or vegan and their median dietary intake of B_12_ was non-significantly lower than non-vegetarians (2.95 vs 3.87 μg/day), while folate consumption in the former group was higher (see online supplementary table S1). Vegetarians had lower serum B_12_ concentrations (median 192 vs 248 pmol/L, p<0.01) but their folate or Hcy concentrations did not vary significantly.

10.1136/bmjopen-2016-011247.supp1 Supplementary table Biochemical indices in vegetarian and vegan women of child-bearing age compared to non-vegetarians

### 

#### Predictors of B_12_, folate and Hcy

**Figure 1:**
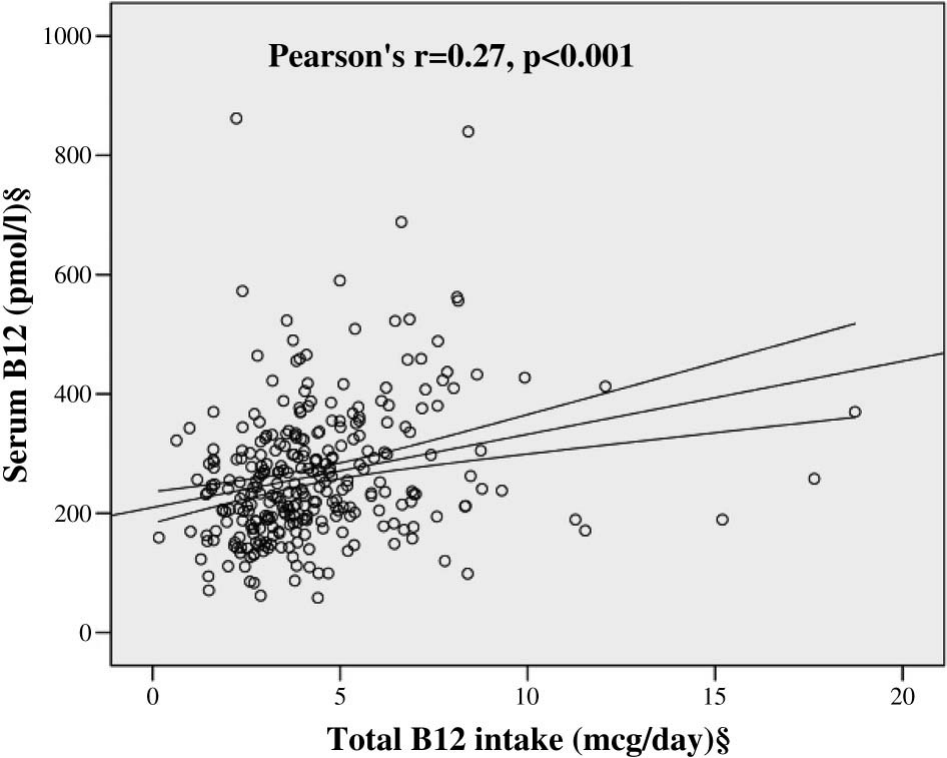
Correlation between daily B_12_ intake and serum B_12_ values. §Log transformed for statistical comparisons.

There was a positive correlation between serum B_12_ values and daily B_12_ intake (Pearson's r=0.27, p<0.001) (figure 1). Simple linear regression analyses of the predictors of B_12_, folate and Hcy are shown in table 3. After adjusting for the likely confounders, daily B_12_ and folic acid intakes were positive predictors of serum B_12_ (β=0.28, p<0.001) and serum folate (β=0.33, p<0.001), respectively. Along with age, serum B_12_, serum folate and B_12_ intake were independent predictors of Hcy, though it would appear that serum folate is strongest based on the value of the β coefficient (table 3).

**Table 3:** Multiple linear regression analysis of predictors of serum B_12_, folate and homocysteine

Variables	Serum B_12_*		Serum folate		Homocysteine*	
	β coefficient	p Value	β coefficient	p Value	β coefficient	p Value
Age	–	NS	–	NS	0.18	0.001
BMI	–	NS	–	NS	–	NS
Smoking	–0.12	<0.05	–	NS	–	NS
Alcohol	–	NS	–	NS	–	NS
Oral contraceptive use	−0.29	<0.001	0.11	<0.05	–	NS
TC:HDL ratio*	–	NS	–	NS	–	NS
Vegetarian or vegan diet	−0.18	<0.01	–	NS	–	NS
B_12_ supplement use	–	NS	Not included	NA	–	NS
Daily B_12_ intake*	0.28	<0.001	Not included	NA	–0.16	0.001
Folic acid supplement use	Not included	NA	0.18	<0.01	–	NS
Daily folate intake*	Not included	NA	0.33	<0.001	–	NS
Serum B_12_*			–	NS	−0.20	<0.001
Serum folate*	–	NS			−0.35	<0.001

*Log transformed for statistical comparison.

–, Tested but not significant in the model;

BMI, body mass index; NA, not applicable; NS, not significant; TC:HDL, total cholesterol:high-density lipoprotein ratio.

#### Relationship between B_12_ intake and associated biomarkers

In total, 5 out of 299 (1.7%) women consumed below the EAR of 1.25 μg/day, but none of them had B_12_ levels <150 pmol/L. Conversely, in the 98.3% of women with ‘adequate’ EAR category for B_12_ consumption, 12.2% of them had serum levels below 150 pmol/L.

**Figure 2:**
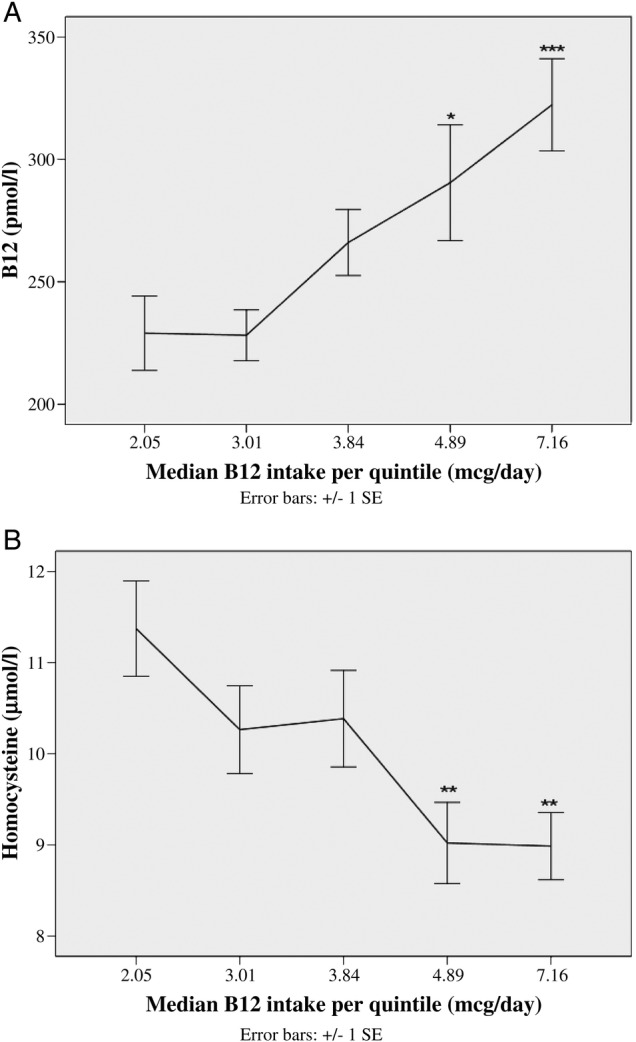
Relationship between B_12_ intake in quintiles and (A) mean serum B_12_ and (B) mean plasma homocysteine concentrations. Mean±SEM values are plotted against the median B_12_ intake in each quintile. One-way analysis of variance (ANOVA) test used to compare the means between the quintiles (after log transformation) and Tukey’s post hoc analysis done. Mean biomarker levels differed as compared with quintile 1 as follows: *p<0.05; **p<0.01; ***p<0.001.

In order to determine the trend of blood B_12_ and Hcy concentrations with increasing intakes of B_12_, we divided the cohort in quintiles of B_12_ intake. The median levels in each quintile and biomarker values are represented in figures 2A, B. Women in quintiles 4 and 5 (median intake 4.9 and 7.1 μg/day, respectively) had significantly higher mean B_12_ and lower mean Hcy concentrations than quintile 1 (291 and 322 vs 229 pmol/L; 9.0 and 9.0 vs 11.4 µmol/L, respectively). Additionally, the Hcy levels appear to level off between quintiles 4 and 5, suggesting that increasing intakes above 5 μg/day is unlikely to provide higher B_12_ at the tissue level (figure 2B). To confirm this, we performed logistic regression to determine the intake threshold at which the odds of hyperhomocysteinemia would reduce significantly after correcting for confounders. Using 0.25 μg increments, a threshold of 4.75 μg/day significantly reduced the odds of Hcy>12 µmol/L (adjusted OR (AOR) 0.35, 95% CI 0.14 to 0.88). When serum B_12_ was added to the model, the significance was lost, suggesting that the influence of dietary B_12_ intake on circulating Hcy is mediated through serum B_12_ values in a folate-replete population (data not shown).

#### Micronutrient intake: comparisons between NDNS 2000/2001 and 2008/2012 data

Comparison of the mean nutrient intake among young women between the two surveys is shown in table 4. The proportion of women consuming below the UK RNI for folate increased in the more recent survey (44% vs 33%, p<0.01) and median intake levels fell by 14% (206 vs 235 pmol/L, p<0.001). There were no significant differences in the B_12_ intake values.

**Table 4:** Comparison between folic acid and B_12_ intakes between the NDNS 2000/2001 and 2008/2011 cohorts

Female 19–39 years	NDNS 2000/2001 cohort	NDNS 2008/2011 cohort
Number	299	395
Folic acid supplement users, n (%)	32 (10.7)	12 (3.0)***,†
Folate intake, diet only (μg/day)	234.9 (175.7, 302.7)‡	205.8 (162.3, 261.2)***
n (%) consuming below UK RNI of folate	99 (33.1)	173 (43.8)**
B_12_ supplement users, n (%)	31 (10.4)	33 (8.4)
B_12_ intake, diet only (μg/day)	3.82 (2.75, 5.01)	3.78 (2.64, 4.84)
n (%) consuming below UK RNI of B_12_	11 (3.7)	24 (6.1)

Significance: **p<0.01; ***p<0.001

†p Values from independent-samples t-test for continuous variables (after log transformation) or χ^2^ test for categorical variables.

‡Median, 25–75th centile in parentheses (all such values).

UK RNI, UK Recommended Nutrient Intake; NDNS, National Diet and Nutrition Survey.

## Discussion

Our study shows contemporary data of B_12_ and folate intakes and serum levels in population-based nutritional surveys involving women of childbearing age, who are representative of the UK population. The key findings are that, despite an apparent adequate daily intake of B_12_, a high proportion of women have B_12_ deficiency and hyperhomocysteinemia.

### Optimum B_12_ intake and biomarker levels

Our data showed positive correlation between B_12_ intake and blood levels with a trend of increasing B_12_ concentration even in intakes up to 7 μg/day, although the corresponding reductions in Hcy levels level off around the intake of 5–7 μg/day. Additionally, the consumption of around 4.75 μg/day was independently associated with a decrease in the odds of hyperhomocysteinemia. Our findings are supportive of the dose–response relationship between intake and blood levels of B_12_ found previously, which showed doubling B_12_ intakes would increase B_12_ concentrations by around 10%.^38^

A daily intake of 4–10 μg has been suggested by other studies to stabilise levels of B_12_, Hcy and the associated MMA in adults,^39^ ^40^ but more evidence is needed before extrapolating these figures to women of childbearing age due to the different requirements and implications should they become pregnant. In a randomised controlled trial where all subjects consumed 8.6 μg/day of B_12_, pregnant women had higher holotranscobalamin (holoTC):total B_12_ ratios than non-pregnant, non-lactating controls, suggesting that such high intakes were required to provide sufficient supply to the developing fetus.^41^ More research is required to decide on the upper limit of B_12_ intake in women before and during pregnancy, as higher B_12_ intake in the third trimester was positively associated with offspring birth weight in high-BMI women (who are already at high risk of having macrosomic babies), although circulating B_12_ levels were not reported here.^42^ There is also no consistent evidence that increased B_12_ intake or supplementation is associated with reduction in the prevalence of subclinical B_12_ deficiency in adults (particularly neurocognitive decline in the elderly).^43^ ^44^ Therefore, until the availability of further studies, our data call for revision of the UK RNI (and EAR) for B_12_ to be at least in line with the current European recommendations (4.5 μg/day).^24^

The estimated B_12_ intake in preconceptional or women in early pregnancy and specifically its relationship with serum levels has not been widely studied. In developing countries, over 50% of women of reproductive age do not meet the RNIs for B_12_^45^ ^46^ and separate surveys from three developing countries (Turkey, Iran and China) found B_12_ deficiency rates of 21–23%.^47–49^ An Australian study on young females found a similar rate to ours (11.4%), albeit using a lower threshold of 120 pmol/L.^50^ As the B_12_ levels fall by around 10% from preconception to early pregnancy,^19^ if we extrapolate our findings, the proportion of pregnant women with B_12_ levels below 150 pmol/L is likely to be much higher. More than a third of vegetarian/vegan women in our study had B_12_ deficiency, which was in keeping with studies from other UK adult population^51^ and elsewhere.^52^ Thus our findings highlight the need for specific advice for the vegetarian/vegan population about potential sources of B_12_, as well as recommending them to have their B_12_ levels checked (and corrected) if they are planning pregnancy.

### Folate intake

Our study shows that consumption of dietary folate has fallen by around 14% in the 10 years between the two surveys and nearly 50% of women are now consuming below the UK RNI. The Scientific Advisory Committee on Nutrition within the UK Food Standards Agency has recommended mandatory folic acid fortification of food products to the UK DOH,^53^ which, if implemented, would increase folate intake and consequently serum levels in the population as it did in the USA.^54^ However, if mandatory fortification does occur, it is possible that improving folate levels can reduce B_12_ levels due to usage of the available B_12_.^55^ In addition, B_12_ deficiency is the strongest driver of Hcy in a folate-replete population.^56^ Therefore fortification of food products with B_12_ together with folate should be considered if there is to be a policy change in the UK or, as a minimum, B_12_ supplements recommended for women in the peri-conceptional period, especially if they are at high risk of deficiency.

The strengths of our study are that this is the first study of its kind from the UK to evaluate B_12_ and folate among women in the preconceptional stage. Extensive data on these women including anthropometry, biochemical markers and dietary information allowed comparison of intake and serum levels adjusted for possible confounders. There were three important limitations of this data set: (1) lack of sufficient biochemical data in the second survey, (2) lack of data on other biomarkers such as holoTC or MMA and (3) lack of haematological and clinical information relevant to the effects of B_12_. Since we were able to compare the nutrient intakes between the two cohorts, which was predictive of serum levels in the earlier survey, we believe that the availability of serum B_12_ in the latter cohort may not necessarily change the findings. Hcy is a readily available marker in clinical practice as opposed to holoTC and MMA. Therefore, our findings are applicable to wider clinical practice. Any future prospective studies involving preconceptional or pregnant women should include more clinical information as well as these biomarkers for a better evaluation of B_12_ status. In conclusion, our study supports revision of the UK RNI to at least match the European recommendations and also calls for assessing maternal B_12_ status in preconception or early pregnancy.
