# Supplementary material for: Vitamin B12 status in women of childbearing age in the UK and its relationship with national nutrient intake guidelines: results from two National Diet and Nutrition Surveys
Source: BMJ Open. 2016 Aug 12;6(8):e011247. doi: 10.1136/bmjopen-2016-011247 (PMC4985863; doi:10.1136/bmjopen-2016-011247)
Supplement: Supplementary figures [file bmjopen-2016-011247supp_figures.pdf]

Supplementary Figures

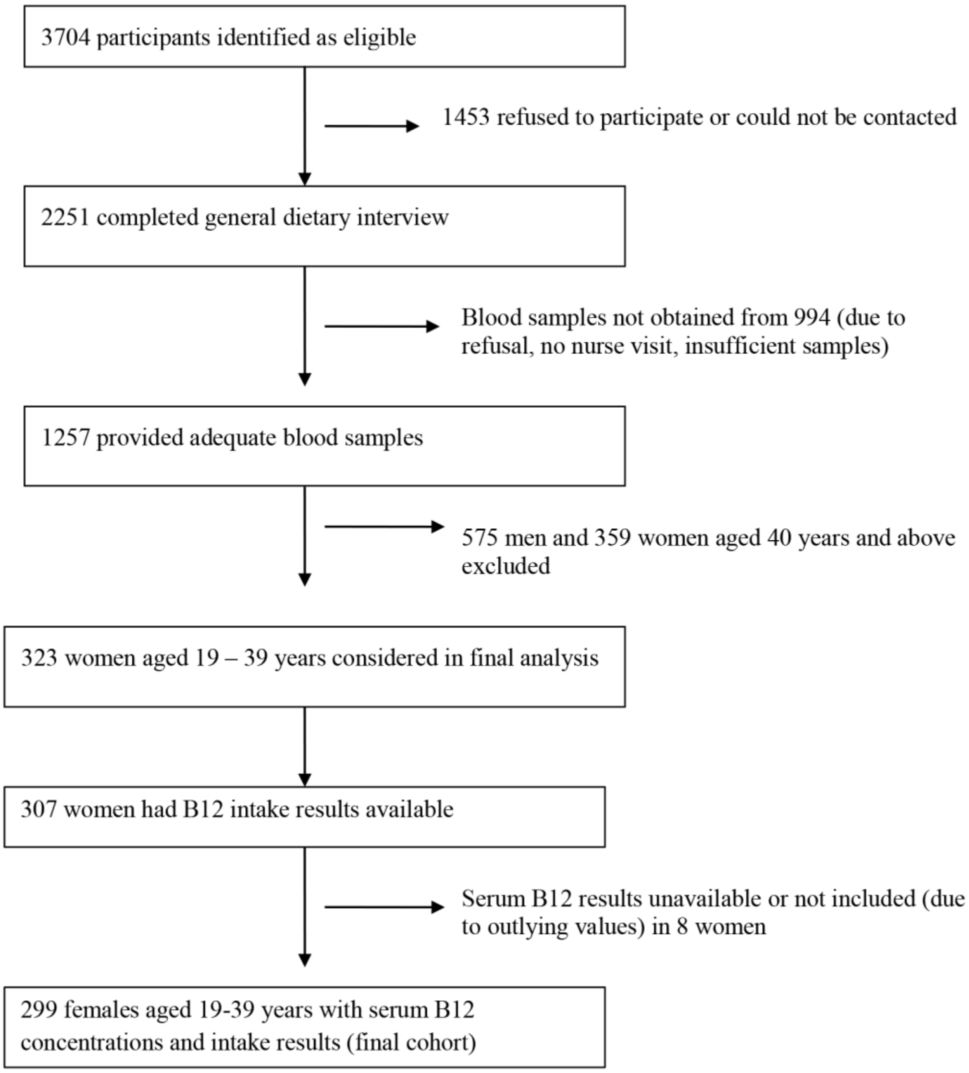

Supplementary Figure 1 Flow diagram illustrating the selection of the final cohort in the NDNS 2000/01 survey

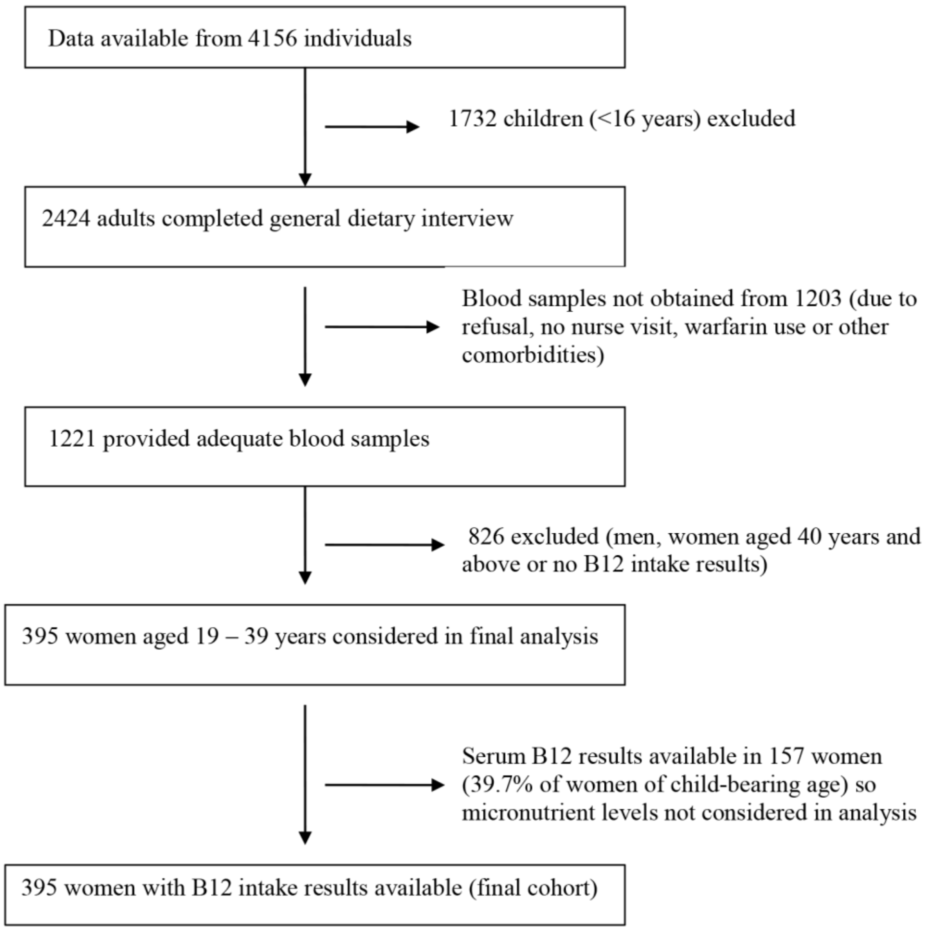

Supplementary Figure 2 Flow diagram illustrating the selection of the final cohort in the NDNS 2008/12 survey
