# Supplementary material for: Vitamin B12 status in women of childbearing age in the UK and its relationship with national nutrient intake guidelines: results from two National Diet and Nutrition Surveys
Source: BMJ Open. 2016 Aug 12;6(8):e011247. doi: 10.1136/bmjopen-2016-011247 (PMC4985863; doi:10.1136/bmjopen-2016-011247)
Supplement: Supplementary table — Biochemical indices in vegetarian and vegan women of child-bearing age compared to non-vegetarians [file bmjopen-2016-011247supp_table.pdf]

Supplementary Table 1 Biochemical indices in vegetarian and vegan women of child-bearing age compared to non-vegetarians

	**Vegetarian / vegan**	**Non-vegetarian**	**P[a]**
Number (%)	26 (8.7)	273 (91.3)	NS
B12 supplements taken, n (%)	5 (19.2)	26 (9.5)	NS
B12 intake, diet only (mcg/day)	2.95 (2.63, 3.96)[b]	3.87 (2.81, 5.09)	NS
B12 intake, diet + supplements (mcg/day)	3.07 (2.65, 4.37)	3.92 (2.86, 5.30)	NS
Serum B12 (pmol/L)	192.0 (138.9, 248.2)	248.3 (190.5, 328.3)	<0.01
B12 insufficiency (<150pmol/L), n (%)	8 (30.8)	28 (10.3)	<0.01
Folic acid supplements taken, n (%)	3 (11.5)	29 (10.6)	NS
Folate intake, diet only (mcg/day)	279.2 (234.6, 324.0)	232.2 (169.3, 292.0)	<0.05
Folate intake, diet + supplements (mcg/day)	284.2 (234.6, 337.5)	233.9 (175.8, 310.2)	NS
Serum folate (nmol/L)	21.8 (18.5, 30.8)	19.2 (13.8, 26.2)	<0.05
Serum folate deficiency (<10nmol/L), n (%)	1 (3.8)	17 (6.3)	NS
Homocysteine (umol/L)	9.60 (7.74, 11.90)	9.24 (7.63, 11.46)	NS
High Hcy (>12 umol/L), n (%)	5 (19.2)	57 (21.3)	NS
High Hcy (>15 umol/L), n(%)	1 (3.8)	23 (8.6)	NS

a: Comparison between vegetarian/vegan and non-vegetarian groups. For categorical variables, Student's t-test was used (after log-transformation); for continuous variables Fisher's exact test was used

b: Median, 25th -75th centile in parentheses (all such values)

NS: not significant
